# Effect of the carbon nanotube surface characteristics on the conductivity and dielectric constant of carbon nanotube/poly(vinylidene fluoride) composites

**DOI:** 10.1186/1556-276X-6-302

**Published:** 2011-04-07

**Authors:** Sónia AC Carabineiro, Manuel FR Pereira, João N Pereira, Cristina Caparros, Vitor Sencadas, Senentxu Lanceros-Mendez

**Affiliations:** 1 Universidade do Porto, Faculdade de Engenharia, Laboratório de Catálise e Materiais (LCM), LSRE/LCM - Laboratório Associado, Rua Dr. Roberto Frias, s/n, 4200-465 Porto, Portugal; 2 Centro/Departamento de Física da Universidade do Minho, Campus de Gualtar, 4710-057 Braga, Portugal

## Abstract

Commercial multi-walled carbon nanotubes (CNT) were functionalized by oxidation with HNO_3_, to introduce oxygen-containing surface groups, and by thermal treatments at different temperatures for their selective removal. The obtained samples were characterized by adsorption of N_2 _at -196°C, temperature-programmed desorption and determination of pH at the point of zero charge. CNT/poly(vinylidene fluoride) composites were prepared using the above CNT samples, with different filler fractions up to 1 wt%. It was found that oxidation reduced composite conductivity for a given concentration, shifted the percolation threshold to higher concentrations, and had no significant effect in the dielectric response.

## Introduction

Carbon nanotubes (CNTs) have attracted particular interest because of their remarkable mechanical and electrical properties [1]. The combination of these properties with very low densities suggests that CNTs are ideal candidates for high-performance polymer composites [2]. In order to increase the application range of polymers, highly conductive nanoscale fillers can be incorporated into the polymeric matrix. As CNTs present high electrical conductivity (10^3^-10^4 ^S/cm), they have been widely used [3]. Therefore, CNT/polymer composites are expected to have several important applications, namely, in the field of sensors and actuators [4]. However, in order to properly tailor the composite material properties for specific applications, the relevant conduction mechanisms must be better understood.

The experimental percolation thresholds for CNT composites results in a wide range of values for the same type of CNT/polymer composites [5], being a deviation from the bounds predicted by the excluded volume theory and a dispersion for the values of the critical exponent (*t*) [6,7]. It was demonstrated that the conductivity of CNT/polymer composites can be described by a single junction expression [8] and that the electrical properties also strongly depend on the characteristics of the polymer matrix [9]. This article explores the effects of nanotubes surface modifications in the electrical response of the composites.

## Experimental

### Preparation and characterization of the modified CNT samples

Commercial multi-walled CNTs (Nanocyl - 3100) have been used as received (sample CNTs). Further details on this material can be found elsewhere [10]. CNTs sample was functionalized by oxidation under reflux with HNO_3 _(7 M) for 3 h at 130°C, followed by washing with distilled water until neutral pH, and drying overnight at 120°C (sample CNTox was obtained). The CNTox material was heat treated under inert atmosphere (N_2_) at 400°C for 1 h (sample CNTox400) and at 900°C for 1 h (sample CNTox900), to selectively remove surface groups. The obtained samples were characterized by adsorption of N_2 _at -196°C, temperature-programmed desorption (TPD) and determination of pH at the point of zero charge (pH_PZC_) from acid-base titration according to the method of the literature [11]. The total amounts of CO and CO_2 _evolved from the samples were obtained by integration of the TPD spectra.

### Composites preparation

Polymer films with thicknesses between 40 and 50 μm were produced by mixing different amounts of CNT (from 0.1 to 1.0%) with *N, N*-dimethylformamide (DMF, Merck 99.5%) and PVDF (Solef 1010, supplied by Solvay Inc., molecular weight = 352 × 10^3 ^g/mol) according to the procedure described previously [9]. Solvent evaporation, and consequent crystallization, was performed inside an oven at controlled temperature. The samples were crystallized for 60 min at 120°C to ensure the evaporation of all DMF solvents. After the crystallization process, the samples were heated until 230°C and maintained at that temperature for 15 min to melt and erase all polymer memory. This procedure produced α-PVDF crystalline phase samples [12].

### Sample characterization

Topography of the samples and CNT distribution was performed by scanning electron microscopy (SEM, FEI - NOVA NanoSEM 200). The dielectric response of the nanocomposites was evaluated by dielectric measurements with a Quadtech 1920. Circular gold electrodes of 5-mm diameter were evaporated by sputtering onto both sides of each sample. The complex permittivity was obtained by measuring the capacity and tan δ in the frequency range of 100 Hz to 100 kHz at room temperature. The volume resistivity of the samples was obtained by measuring the characteristic *I*-*V *curves at room temperature using a Keithley 6487 picoammeter/Voltage source.

## Results and discussion

### Characterization of CNT samples

**Figure 1:**
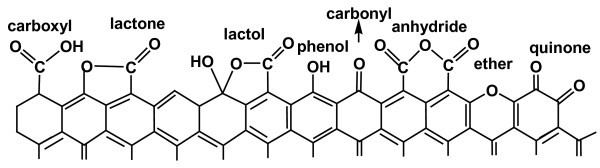
**Acidic and basic groups on CNT's surface**.

Oxidations with HNO_3 _originate materials with large amounts of surface acidic groups, mainly carboxylic acids and, to a smaller extent, lactones, anhydrides, and phenol groups [10,13,14]. These oxygenated groups (Figure 1) are formed at the edges/ends and defects of graphitic sheets [15]. The different surface-oxygenated groups created upon oxidizing treatments decompose by heating, releasing CO and/or CO_2_, during a TPD experiment. As this release occurs at specific temperatures, identification of the surface groups is possible [10,13,14]. It is well known that CO_2 _formation results from the decomposition of carboxylic acids at low temperature, and lactones at higher temperature; carboxylic anhydrides originate both CO and CO_2_; phenols and carbonyl/quinone groups produce CO [10,13,14].

**Figure 2:**
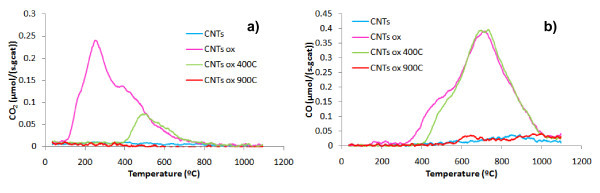
**TPD spectra of the CNT samples before and after the oxidizing treatments**: CO_2 _**(a) **and CO **(b) **evolution.

Figure 2 shows the TPD spectra of the CNT before and after the different treatments. It is clear that the treatment with HNO_3 _produces a large amount of acidic oxygen groups, such as carboxylic acids, anhydrides, and lactones, which decompose to release CO_2_. Part of these groups (carboxylic acids) is removed by heating at 400°C. A treatment at 900°C removes all the groups, so that the obtained sample is similar to the original. The total amounts of CO and CO_2 _evolved from the samples, obtained by integration of the TPD spectra, are presented in Table 1.

**Table 1:** BET surface areas obtained by adsorption of N_2 _at -196°C and amounts of CO_2 _and CO obtained by integration of areas under TPD spectra

Sample	CNTs	CNTox	CNTox400	CNTox900
BET surface area (m^2^/g)	254	400	432	449
pH_PZC_	7.3	4.2	6.9	7.4
CO_2 _(μmol/g)	70	778	230	24
CO (μmol/g)	193	1638	1512	204
CO/CO_2_	2.76	2.11	6.57	8.50

All the samples release higher amounts of CO than CO_2 _groups (Table 1). The CNTox sample has the highest amount of surface oxygen. This sample also presents the lowest ratio CO/CO_2 _and the lowest value of pH_PZC_, indicating that this is the most acidic sample. CNTox900 presents the highest CO/CO_2 _ratio, suggesting the less-acidic characteristics, which matches well with the pH_PZC _results (Table 1). The acidic character of the samples decreases by increasing the thermal treatment temperature, since the acidic groups are removed at lower temperatures than neutral and basic groups, as seen in previous studies [10,13,14].

The CNT samples have N_2 _adsorption isotherms of type II (not shown), as expected for non-porous materials [16]. The surface areas of the samples, calculated by the BET method (S_BET_), are included in Table 1. It can be observed that the oxidation treatments lead to an increase of the specific surface area. This occurs because the process opens the endcaps of CNTs and creates sidewall openings [17]. The specific surface areas of the samples slightly increase as the thermal treatment temperature increases, since carboxylic acids and other groups, introduced during oxidation, are removed.

### Composites processing and characterization

**Figure 3:**
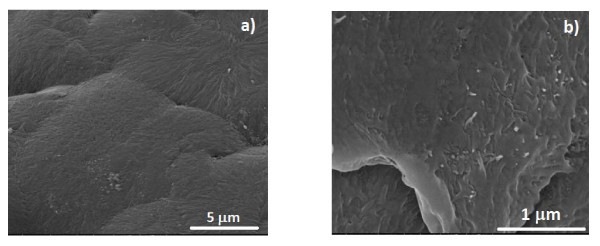
**SEM images for the PVDF@ CNTox400 composites (for 0.2% CNTox400)**: **(a) **surface image showing the spherulitic microstructure of the polymer and **(b) **fracture image showing the dispersion of the CNT into the bulk of the polymeric matrix.

The morphology and fiber distribution of the composite samples were analyzed by SEM to evaluate the CNT dispersion in the polymeric matrix and determine how the composites influence the polymer crystallization microstructure. Figure 3 shows the SEM images for the PVDF/CNT composites. The main relevant microstructural feature of the composite is that the CNT are randomly distributed into the polymeric matrix. The spherulitic structure characteristic of the pure PVDF is still present in all the composites samples [12,18].

**Figure 4:**
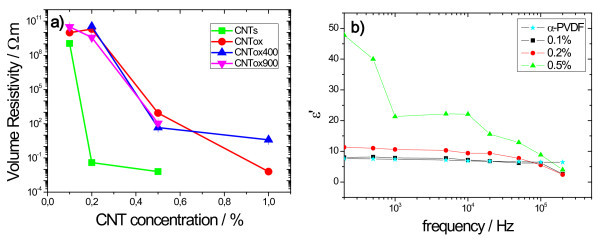
**Electrical response of the PVDF/CNT nanocomposites**: **(a) **Volume resistivity of the PVDF/CNT nanocomposites for the different functionalized CNTs; **(b) **dielectric constant at room temperature and 10 kHz for the PVDF/CNT original composites.

CNT agglomerates are nevertheless more often observed for the CNTox composites samples, especially for the ones treated at the highest temperatures. With respect to the electrical properties, oxidation reduces the composite conductivity for a given concentration and shifts the percolation threshold to higher concentrations (Figure 4). This behavior is mainly due to the reduction of the surface conductivity of the CNTs due to the oxidation process [8], and is similar for all the functionalized composites. Further, the increase of surface area due to the functionalization treatment certainly causes surface defects on the CNTs that also reduced electrical conductivity. The increase of agglomerations for the treated samples should not have, on the other hand, a large influence in the electrical response [8]. A change of several orders of magnitude of the electrical resistivity with increasing CNTs concentration was observed for all samples, indicating a percolative behavior of the nanocomposites. In general, both in surface (not shown) and in bulk resistivity (Figure 4a), the percolation threshold appears between 0.2 wt.% for the original CNT samples and shifts to 0.5 wt.% CNTs for the functionalized nanocomposites.

Dielectric measurements show that the incorporation of the CNT in the PVDF matrix but leads to a gradual increase of the dielectric constant (ε') as the amount of the filler is increased (Figure 4b). The increase of the ε' is larger for the pristine CNT. A maximum for the 0.5% pristine CNT sample with ε' 22 at a frequency of 10 kHz at room temperature was found, whereas for the functionalized nanocomposites the value is 16. The frequency behavior of the dielectric permittivity is similar to the one obtained for the pure polymer, except for an increase of the low frequency dielectric constant and dielectric loss (not shown) with increasing CNT loading due to interfacial polarization effects (Figure 4b). No noticeable differences have been observed for the different oxidation treatments in terms of the dielectric response. In a previous study [19], it was demonstrated that an increase in the dielectric constant is related with the formation of a capacitor network.

## Conclusions

The effect of surface modifications of multi-walled CNTs on the electrical response of CNT/PVDF nanocomposites has been investigated. The main effect of oxidation is a reduction of the composite conductivity for a given concentration and a shift of the percolation threshold to higher concentrations. On the other hand, no significant differences have been observed between the nanocomposites prepared with the different functionalized CNTs. The reduction of the electrical surface conductivity of the CNT due to the oxidation process, together with an increase of the surface area and defect formation, is at the origin of the observed effects.

## Abbreviations

CNT: carbon nanotubes; DMF: *N, N*-dimethylformamide; SEM: scanning electron microscopy.

## Competing interests

The authors declare that they have no competing interests.

## Authors' contributions

SACC performed the functionalisation and characterisation of carbon nanotubes samples and drafted the manuscript. JNP, CP, and VS participated in the nanocomposite samples processing, experimental measurements, analysis and interpretation of the results. MFRP and SL-M conceived and coordinated the research work and carried out analysis and interpretation of the experimental results. All authors read and approved the final manuscript.
